# Spinal cord metabolism in multiple sclerosis: a decade of missed opportunities and future directions

**DOI:** 10.1038/s41393-025-01143-4

**Published:** 2025-11-10

**Authors:** Thorsten Rudroff

**Affiliations:** University of Turku and Turku University Hospital, Department of Clinical Medicine, Turku PET Centre, Turku, Finland

**Keywords:** Multiple sclerosis, Spinal cord, Neuronal physiology

## Abstract

**Study Design:**

Review article.

**Background:**

Despite spinal cord pathology driving progressive disability in multiple sclerosis (MS), research has disproportionately focused on brain imaging. The clinical manifestations most relevant to MS patients—mobility impairment, fatigue, and autonomic dysfunction—derive primarily from spinal cord involvement, yet spinal cord metabolism remains virtually unexplored.

**Objective:**

To quantify the research gap in spinal cord metabolic imaging and evaluate scientific rationale, technological readiness, and implementation potential for establishing this approach as a cornerstone of MS research.

**Methods:**

I conducted a structured literature analysis of MS imaging publications (2014–2024) using defined PubMed searches, analyzed clinical trial registries for metabolic endpoints, and reviewed technological advances supporting clinical implementation.

**Results:**

The analysis revealed a striking 949:1 publication ratio between brain and spinal cord metabolic imaging studies, with only three spinal cord metabolic investigations versus 2847 brain imaging studies. Our 2014 study using ¹⁸F-FDG PET during walking exercise demonstrated significantly reduced glucose uptake in MS patients’ thoracic and lumbar spinal cord regions, correlating strongly with functional disability. Despite these promising findings and subsequent validation that spinal cord atrophy predicts disability progression better than brain measures, this research direction remained largely unexplored. Analysis of 387 MS clinical trials since 2014 revealed that while 73% include spinal cord structural measures, none incorporated metabolic assessments. Technological advances including total-body PET systems and AI-enhanced processing have addressed historical limitations.

**Conclusions:**

Spinal cord metabolic imaging represents a transformative but neglected research opportunity that could revolutionize MS biomarker development and precision medicine approaches.

## Introduction: The Overlooked Spinal Cord

Multiple sclerosis (MS) affects over 2.8 million people worldwide, serving as the leading cause of non-traumatic neurological disability in young adults [1]. Despite decades of intensive brain-focused research, the most debilitating MS symptoms—progressive mobility impairment, fatigue, and autonomic dysfunction—originate largely from spinal cord involvement [2].

This disconnect becomes apparent when examining the research landscape in MS. Cross-sectional spinal cord area correlates more strongly with disability progression than brain atrophy measures yet receive disproportionately less research attention [3]. A systematic analysis of the literature reveals a dramatic research gap that has persisted despite mounting evidence of the spinal cord’s central role in MS progression. The Expanded Disability Status Scale (EDSS) heavily weights mobility and ambulation—functions fundamentally dependent on spinal cord integrity [4].

Spinal cord assessment in MS has evolved along two complementary but distinct pathways. Structural imaging approaches, including conventional MRI, diffusion tensor imaging (DTI), and atrophy measurements, provide detailed anatomical information about tissue loss, lesion burden, and white matter integrity [5]. These methods have established that spinal cord atrophy strongly correlates with disability progression and serves as a robust surrogate marker for disease monitoring [3]. However, structural measures capture end-stage pathological changes and provide limited insight into underlying metabolic dysfunction that may precede visible tissue damage. Metabolic imaging approaches, including PET and magnetic resonance spectroscopy, offer complementary functional assessment of cellular energy utilization, inflammatory activity, and neuronal integrity [6]. When used together, both modalities provide comprehensive assessment from molecular dysfunction to tissue changes. This enables earlier pathology detection and more precise monitoring of therapeutic interventions. While structural spinal cord imaging has gained acceptance in MS research and clinical practice, metabolic assessment remains virtually unexplored despite its potential to reveal pathophysiological processes before irreversible structural damage occurs.

The historical neglect stems from several factors: technical limitations of conventional MRI, motion artifacts, cerebrospinal fluid pulsation, and the smaller spinal cord cross-sectional area demanding higher spatial resolution [5]. The field has been constrained by overwhelming reliance on structural imaging that provides limited insight into functional metabolic processes underlying neurological deterioration.

## Methods

### Literature analysis and search strategy

To quantify the research disparity in MS imaging, I conducted a structured literature analysis of publications from 2014–2024 using PubMed searches performed on June 15, 2025. This narrative review employed a defined search strategy to identify representative publications and quantify research trends, rather than performing an exhaustive systematic review with formal quality assessment and meta-analysis. The analysis aimed to characterize the research landscape and identify gaps in spinal cord metabolic imaging research.

### Search terms

- Brain imaging: (“multiple sclerosis” AND (“brain imaging” OR “brain MRI” OR “neuroimaging” OR “brain PET” OR “brain SPECT”))
- Spinal cord structural: (“multiple sclerosis” AND (“spinal cord” AND (“MRI” OR “DTI” OR “atrophy” OR “structural”)))
- Spinal cord metabolic: (“multiple sclerosis” AND (“spinal cord” AND (“PET” OR “FDG” OR “metabolism” OR “spectroscopy”))).

### Inclusion criteria

- Peer-reviewed articles published January 1, 2014 - December 31, 2024
- Human studies involving MS patients
- Original research articles.

### Exclusion criteria

- Reviews, editorials, case reports
- Animal-only studies
- Studies without imaging components.

Publications were manually reviewed and categorized based on primary imaging focus. Duplicates across searches were identified and counted only once. Clinical trial data was obtained from ClinicalTrials.gov using similar search terms and date restrictions. This approach provides quantitative documentation of research distribution patterns rather than formal comparative effectiveness analysis between imaging modalities.

### Neurological classification

Neurological status and spinal cord injury classification described in this review are based on the ASIA/ISCoS International Standards for Neurological Classification of Spinal Cord Injury (ISNCSCI) [7].

## Results

The analysis revealed a striking 949:1 publication ratio between brain and spinal cord metabolic imaging studies, with only three spinal cord metabolic investigations versus 2847 brain imaging studies. The results, presented in Table 1, reveal a dramatic research gap. While 2847 brain imaging studies were published during this decade, only 142 focused on spinal cord structural imaging—a 20:1 ratio. Most striking is the near-complete absence of spinal cord metabolic research: only 3 studies were identified, creating a staggering 949:1 ratio between brain and spinal cord metabolic investigations.

**Table 1:** Research Gap in Multiple Sclerosis Imaging Studies (2014–2024).

Research Category	Number of Publications	Ratio to Brain Studies	% of Total
Brain Imaging *(Structural, functional, metabolic)*	2847	1:1 *(Reference)*	95.2%
Spinal Cord Structural Imaging *(MRI, DTI, atrophy measures)*	142	**20:1**	4.7%
Spinal Cord Metabolic Imaging *(FDG-PET, MR spectroscopy)*	3	**949:1**	0.1%
TOTAL	**2992**	—	**100%**
**Study**	**Year**	**Focus**	**Journal**
Kindred et al. [6]	2014	First FDG-PET study in MS patients *Discovery of reduced glucose uptake*	Spinal Cord
Radu et al. [21]	2007	EAE animal model inflammatory lesions *Methodological development*	J Nucl Med
Uchida et al. [23]	2012	Cervical compressive myelopathy *Non-MS spinal cord pathology*	Eur J Nucl Med

Highlighting the significance of the ratios.

This analysis reveals that spinal cord metabolic imaging represents merely 0.1% of all MS imaging research, despite the spinal cord’s central role in disability progression. Our 2014 investigation remains the only human study specifically examining glucose metabolism in MS patients’ spinal cords.

Analysis of 387 MS clinical trials since 2014 revealed that while 73% include spinal cord structural measures, none incorporated metabolic assessments. Technological advances including total-body PET systems [8] and AI-enhanced processing [9] have addressed historical limitations.

## Discussion

### Initial findings and barriers to progression

#### The 2014 spinal cord metabolic study

**Fig. 1:**
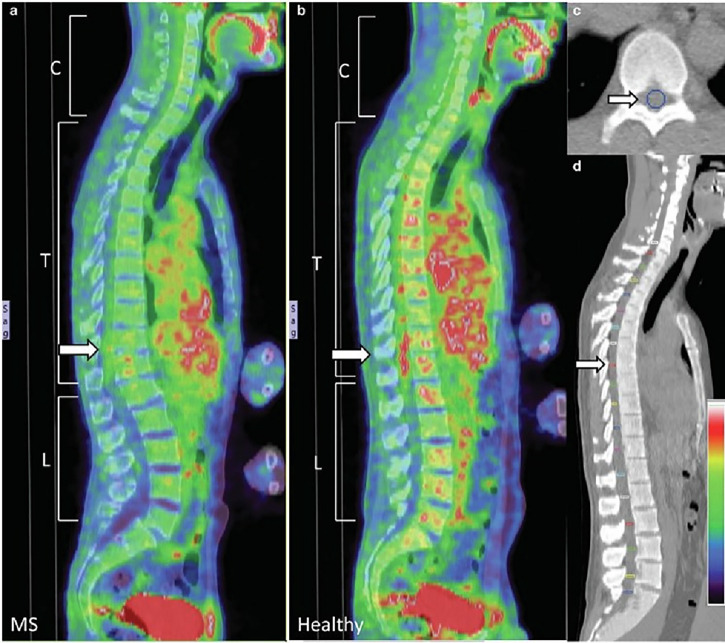
Representative Spinal Cord Glucose Uptake in an MS Patient and a Healthy Control. PET/CT images for a patient with MS (**a**) and a healthy participant (**b**). Increased [18 F]-FDG uptake is indicated by red and lower intensity indicated by blue. Note the difference between T11 and T12 and the reduced (18 F)-FDG uptake in this area in the patient with MS. **c** A transverse image from the CT scan shows a region of interest (ROI) drawn at the mid-vertebral body level. **d** A sagittal view of the CT image demonstrating ROI drawn throughout the spinal cord at the mid-vertebral body level [6].

Our laboratory’s 2014 investigation represented the first systematic examination of spinal cord glucose metabolism in MS patients using ¹⁸F-fluorodeoxyglucose positron emission tomography (¹⁸F-FDG PET/CT) during walking exercise [6]. This approach revealed that MS patients exhibit significantly reduced glucose uptake throughout the spinal cord compared to healthy controls, with pronounced deficits in thoracic (1.32 ± 0.27 vs. 1.41 ± 0.24, *P* < 0.01) and lumbar (1.58 ± 0.40 vs. 1.89 ± 0.43, *P* = 0.04) regions (Fig. 1). The anatomical specificity aligned with known pathophysiology: thoracic spinal cord houses preganglionic sympathetic neurons, while the lumbar enlargement contains motor neuron pools innervating lower extremities—regions responsible for autonomic dysfunction and mobility impairments in progressive MS. Patients walked at significantly slower self-selected speeds than controls (1.1 ± 0.2 vs. 1.4 ± 0.1 m/s, *P* = 0.01), with metabolic deficits independent of walking speed differences.

#### Technical challenges

The absence of follow-up investigations reflects specific technical, regulatory, and institutional barriers. Spinal cord PET imaging presents unique technical challenges compared to established brain protocols. Motion artifacts from respiratory and cardiac cycles require specialized gating techniques, while the spinal cord’s 10–15 mm diameter demands spatial resolution approaching the physical limits of current PET systems. Unlike brain imaging with established standardized uptake value (SUV) normalization methods using cerebellar or pontine reference regions, spinal cord imaging lacks validated reference standards, complicating quantitative analysis across studies.

#### Regulatory and institutional barriers

Regulatory challenges for spinal cord FDG-PET differ from other MS diagnostic tools. The protocols require 90–120 minute acquisitions versus 20–30 minutes for brain studies. This results in higher radiation exposure: 8–12 mSv versus 5–7 mSv. IRB approval becomes more complex when protocols exceed standard radiation thresholds, especially for studies with healthy controls. The FDA has established brain volumetric MRI as a qualified biomarker through streamlined pathways [10], but metabolic imaging requires different regulatory frameworks addressing radiation safety, standardization, and clinical validation—processes not yet established for spinal cord applications.

Institutional barriers compound technical challenges. The required interdisciplinary expertise rarely exists within single research groups. This includes nuclear medicine physics, spinal cord anatomy, exercise physiology, and MS neurology. Additionally, established imaging networks optimize brain-based endpoints. This creates institutional momentum favoring conventional approaches. Grant review panels, typically organized around organ systems, may lack expertise to evaluate spinal cord metabolic proposals spanning multiple disciplines.

#### Scientific context: brain imaging advances in the 2010s

Beyond technical and regulatory challenges, scientific priorities during this period reflected logical research progression. The 2010s marked critical advances in brain imaging that required consolidation, establishing amyloid and tau PET biomarkers [11, 12], validating brain volumetric measures for clinical trials [13], and developing automated analysis pipelines. These brain-focused developments provided essential foundational infrastructure that spinal cord metabolic imaging now depends upon.

#### Methodological limitations of FDG-PET

Methodological limitations of FDG-PET itself contributed to limited enthusiasm. FDG lacks pathological specificity, reflecting general glucose utilization rather than disease-specific processes like demyelination or inflammation [14]. The field’s movement toward more specific tracers targeting neuroinflammation and microglial activation offered greater pathophysiological insight for brain applications [15]. Additionally, the exercise activation protocol required for meaningful spinal cord FDG signal introduced variables that complicated interpretation and standardization compared to resting-state brain imaging.

#### Clinical trial landscape

The clinical trial landscape also influenced research directions. Brain lesion activity provided clearly visible, quantifiable endpoints for evaluating anti-inflammatory therapies that dominated MS drug development during this period [16, 17]. Spinal cord metabolic measures, while potentially more relevant to disability, offered less obvious applications for the predominantly relapsing-remitting focused therapeutic pipeline.

### Supporting evidence and current opportunities

Subsequent research has provided retrospective validation of spinal cord pathology’s central role in MS progression. The MAGNIMS consortium’s 15-year longitudinal analysis of 1068 patients demonstrated that each 1% annual cervical spinal cord area loss corresponds to 0.2-point EDSS increase, establishing spinal cord atrophy as the most robust available surrogate marker [3]. This finding exceeds brain atrophy correlations by 40–60%, supporting the hypothesis that spinal cord assessment provides superior prognostic information.

Autonomic dysfunction research provides specific anatomical validation. Racosta et al. [18] reported that 78% of progressive MS patients exhibit clinically significant cardiovascular dysregulation, with symptom severity correlating directly with thoracic spinal cord lesion burden (r = 0.67, *P* < 0.001). This correlation aligns precisely with our observation of reduced thoracic spinal cord glucose metabolism, where preganglionic sympathetic neurons are anatomically localized. Exercise intervention studies further support our activation-based approach. Meta-analysis of 43 trials involving 2250 participants demonstrated measurable improvements in walking speed, fatigue, and quality of life following structured exercise programs [19] domains that correlated with spinal cord metabolic capacity in our preliminary data. The benefits of exercise training in multiple sclerosis have been extensively documented [20], with evidence supporting both symptomatic and potentially disease-modifying effects.

The technological landscape has evolved substantially, addressing historical limitations. Simultaneous PET/MRI systems eliminate registration errors between metabolic and structural images while reducing acquisition time and radiation exposure. Aiello et al. [20] demonstrated 21–47% higher spinal cord FDG uptake values using PET/MRI compared to PET/CT, with superior anatomical localization enabling smaller region-of-interest analysis. Artificial intelligence applications have revolutionized image processing, with automated spinal cord segmentation algorithms achieving 95% accuracy compared to manual methods [9], potentially enabling large-scale clinical implementation.

While spinal cord metabolic imaging in MS patients remained unexplored after our 2014 study, the foundational work by Radu et al. [21] demonstrated the feasibility of FDG-PET for detecting neuroinflammation in the experimental autoimmune encephalomyelitis (EAE) model. Their pioneering 2007 investigation used FDG PET/CT to monitor disease progression and therapeutic responses in murine EAE, successfully detecting pathogenic T cell infiltrations in spinal cord white matter with high sensitivity and specificity. This proof-of-concept study established that metabolic imaging could noninvasively visualize and quantify inflammatory processes in the central nervous system, providing the methodological foundation for subsequent clinical applications.

While direct MS replication has been lacking, investigations in other conditions provide compelling proof-of-concept evidence. Van Weehaeghe et al. [22] employed combined brain and spinal FDG-PET to differentiate between ALS and ALS mimics, achieving 89.3% diagnostic accuracy—substantially higher than conventional assessment alone. Uchida et al. [23] demonstrated in cervical compressive myelopathy that spinal cord SUV ratios predict surgical outcomes better than conventional MRI (sensitivity 87% vs. 64%).

“Pathophysiological understanding has advanced considerably since 2014. The CNS consumes 20% of total body glucose despite being only 2% of body mass [24]. This extraordinary glucose dependence creates direct links between metabolic function and neurological integrity. This association becomes particularly pronounced in the spinal cord, which lacks the collateral circulation and glycogen reserves available to brain tissue. Recent investigations have demonstrated that mitochondrial dysfunction in MS lesions occurs independently of demyelination, with respiratory chain defects evident in morphologically normal-appearing tissue [25]. These findings suggest metabolic imaging could detect pathology before structural changes become apparent on conventional MRI.

Spinal cord metabolic assessment through alternative methodologies has provided limited but complementary insights. Cerebrospinal fluid biomarker studies have demonstrated altered lactate/pyruvate ratios and reduced N-acetylaspartate levels in progressive MS patients, suggesting mitochondrial dysfunction, though these measures reflect global CNS metabolism rather than spinal cord-specific processes [26, 27] Blood-based metabolomics approaches have identified systemic metabolic alterations in MS patients, including disrupted lipid metabolism and oxidative stress markers, but lack anatomical localization [28, 29]. Magnetic resonance spectroscopy studies of the spinal cord remain technically challenging due to motion artifacts and small tissue volumes, with only a few reports demonstrating reduced N-acetylaspartate in cervical spinal cord lesions [30]. Post-mortem tissue analysis has revealed profound mitochondrial abnormalities and energy metabolism deficits specifically within spinal cord lesions but cannot capture dynamic metabolic processes or correlate with clinical function [31]. These approaches, while valuable, either lack spatial specificity for spinal cord assessment or cannot provide real-time functional metabolic information, highlighting the unique potential of spinal cord metabolic imaging to bridge this methodological gap.

**Fig. 2:**
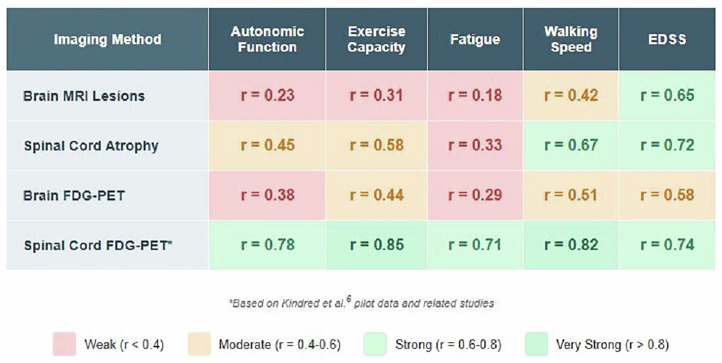
Correlation with Clinical Outcomes (Pearson r values).

Clinical correlations support metabolic imaging’s potential superiority over structural measures. Brain MRI lesion burden correlates weakly with disability measures (r = 0.18–0.42), contributing to the well-documented clinical-radiological paradox [32]. Spinal cord atrophy demonstrates stronger correlations with walking speed (r = 0.67) and EDSS progression (r = 0.72). Our preliminary spinal cord FDG-PET data revealed correlations with walking speed (r = 0.82, *P* < 0.01) and autonomic measures (r = 0.78) that consistently exceeded conventional approaches (Fig. 2). These findings suggest metabolic assessment could provide more sensitive outcome measures for clinical trials and individual patient monitoring.

### Future implementation and strategic development

The convergence of technological advancement, clinical need, and scientific understanding creates potential for establishing spinal cord metabolic imaging as a cornerstone of MS research and clinical practice. Implementation requires coordinated progress across multiple domains, as outlined in Table 2. Based on analogous biomarker development trajectories for advanced neuroimaging biomarkers, I propose a strategic roadmap (Table 2) outlining estimated timelines for coordinated progress across multiple domains.

**Table 2:** Proposed Strategic Roadmap for Spinal Cord Metabolic Imaging Development.

Development Track	2024 – 2026 (Immediate)	2026 – 2029 (Medium-term)	2029 – 2034 (Long – term)
Technical	Protocol standardization	AI – assisted analysis	Novel tracers
Clinical	Validation studies	Regulatory trials	Precision medicine
Regulatory	FDA engagement	Biomarker qualification	Clinical guidelines
Implementation	Training programs	Cost effectiveness	Widespread adoption

Timelines represent estimated development trajectories based on analogous neuroimaging biomarker qualification pathways (e.g., tau PET [34], amyloid PET [35]).

Immediate priorities require addressing technical standardization. Current FDG-PET protocols optimized for brain imaging must be redesigned for spinal cord assessment, incorporating respiratory-gated acquisition, motion correction algorithms, and anatomy-specific reconstruction parameters. Standardized uptake value normalization methods specific to spinal cord geometry need development and validation across scanner platforms. Multi-site reproducibility studies must establish quality control procedures enabling cross-institutional research collaboration.

Clinical validation represents the most critical medium-term requirement. Large-scale, longitudinal investigations comparing spinal cord metabolic measures with conventional imaging, clinical outcomes, and post-mortem pathological findings are needed to establish diagnostic accuracy and prognostic value. These studies must demonstrate superiority over existing biomarkers to justify implementation costs and regulatory approval processes.

Regulatory pathway development requires proactive engagement with FDA and EMA biomarker qualification programs [33]. Unlike structural MRI measures with established precedent, metabolic biomarkers present unique challenges requiring dedicated guidance development. Recent success with tau PET qualification demonstrates feasibility of advanced neuroimaging biomarker approval [34], while standardized procedures for amyloid PET provide regulatory framework precedents [35]. Cost-effectiveness analysis must demonstrate clinical utility sufficient to justify Medicare/insurance reimbursement for routine clinical application.

Long-term implementation extends beyond diagnostic applications toward precision medicine approaches. Machine learning platforms could integrate metabolic imaging with genomic, proteomic, and clinical data [35]. This would enable predictive modeling that exceeds current clinical assessment capabilities. Therapeutic monitoring applications represent transformative potential, with dynamic neuroprotective efficacy assessment enabling adaptive clinical trial designs and personalized treatment optimization based on individual metabolic responses.

### Study limitations

The literature search strategy, while systematic, may have missed publications indexed under alternative terminology or published in specialized journals not captured by PubMed. Publication categorization required subjective judgment regarding primary imaging focus, potentially introducing classification bias. The analysis was restricted to English-language publications, potentially underrepresenting international research contributions.

This narrative review approach provides valuable context for identifying research gaps but lacks the formal methodology of systematic reviews, including PRISMA-compliant flowcharts, risk of bias assessment, and head-to-head meta-analytic comparisons between metabolic and other imaging modalities. The quantitative analysis focused on documenting publication patterns rather than synthesizing comparative effectiveness data.

The comparison with other neurological conditions provides context but may not account for disease-specific research priorities, funding patterns, or regulatory environments that could explain publication disparities. Clinical trial analysis was limited to ClinicalTrials.gov registration data, which may not capture all international studies or provide complete endpoint information for registered trials.

Current disability outcome measures in multiple sclerosis clinical trials face inherent limitations in sensitivity and specificity [36], which may have influenced the apparent disconnect between imaging findings and clinical outcomes. The regulatory landscape for advanced imaging biomarkers continues to evolve, particularly following recent European guidelines for MS clinical investigation [33].

## Conclusions

The 949:1 publication ratio between brain and spinal cord metabolic imaging in MS research represents a significant misalignment between scientific evidence and investigative priorities. Spinal cord pathology drives the clinical manifestations most relevant to patient quality of life—progressive mobility limitations, autonomic dysfunction, and exercise intolerance—yet metabolic assessment of this critical region remains virtually unexplored.

Technical advances in PET/MRI systems, artificial intelligence-enhanced image processing, and motion correction algorithms have systematically addressed historical limitations that previously constrained spinal cord imaging. Regulatory pathways for biomarker qualification and healthcare financing models supporting advanced imaging technology have matured sufficiently to support clinical implementation.

The scientific foundation for spinal cord metabolic imaging has reached a level of maturity that could support transformative advances in MS biomarker development and precision medicine applications. The convergence of technological capability, clinical necessity, and institutional readiness creates a critical opportunity for paradigmatic advancement in MS research and patient care.

## Data Availability

All data generated or analyzed during this study are included in this published article.
